# Adaptive brain activity changes during tongue movement with palatal coverage from fMRI data

**DOI:** 10.1038/s41598-021-93332-3

**Published:** 2021-07-06

**Authors:** Yuka Inamochi, Kenji Fueki, Nobuo Usui, Masato Taira, Noriyuki Wakabayashi

**Affiliations:** 1 Removable Partial Prosthodontics, Graduate School of Medical and Dental Sciences, Tokyo Medical and Dental University (TMDU), 1-5-45 Yushima, Bunkyo-ku, Tokyo, 113-8549 Japan; 2 Biointerfaces Unit, Institute of Innovative Research, Tokyo Institute of Technology, S3-12 2-12-1 Ookayama, Meguro-ku, Tokyo, 152-8550 Japan; 3 Department of Cognitive Neurobiology, The Center for Brain Integration Research, Graduate School of Medical and Dental Sciences, Tokyo Medical and Dental University (TMDU), 1-5-45 Yushima, Bunkyo-ku, Tokyo, 113-8549 Japan

**Keywords:** Motor control, Dental diseases

## Abstract

Successful adaptation to wearing dentures with palatal coverage may be associated with cortical activity changes related to tongue motor control. The purpose was to investigate the brain activity changes during tongue movement in response to a new oral environment. Twenty-eight fully dentate subjects (mean age: 28.6-years-old) who had no experience with removable dentures wore experimental palatal plates for 7 days. We measured tongue motor dexterity, difficulty with tongue movement, and brain activity using functional magnetic resonance imaging during tongue movement at pre-insertion (Day 0), as well as immediately (Day 1), 3 days (Day 3), and 7 days (Day 7) post-insertion. Difficulty with tongue movement was significantly higher on Day 1 than on Days 0, 3, and 7. In the subtraction analysis of brain activity across each day, activations in the angular gyrus and right precuneus on Day 1 were significantly higher than on Day 7. Tongue motor impairment induced activation of the angular gyrus, which was associated with monitoring of the tongue’s spatial information, as well as the activation of the precuneus, which was associated with constructing the tongue motor imagery. As the tongue regained the smoothness in its motor functions, the activation of the angular gyrus and precuneus decreased.

## Introduction

The tongue plays important roles in mastication^1^, swallowing^2^, and articulation^3^. In chewing, tongue movement is involved in bolus formation, retention, and feeding^4^. In prosthetic treatments, covering the palate with removable dentures disturbs oral function, including speech^5^, tactile sensation, gustation^6,7^, and swallowing^8^. Therefore, wearing palatal plates may impair tongue motor control. It is thus essential for individuals whose dentures cover the palate to adapt to wearing the dentures, especially while speaking or masticating. Nevertheless, neurophysiological mechanisms associated with adaptation to palatal coverage have not been examined.

We previously observed the adaptation process to palatal coverage, and investigated the temporal changes in masticatory performance and brain activity while chewing with the palate covered^9^. Oral environment changes caused by the palate being covered impaired masticatory performance, but chewing ability fully recovered by day 7 post-insertion. Brain activation in the face primary sensorimotor cortex and putamen decreased immediately post-insertion and fully recovered to pre-insertion levels day 7. Subjects wore the palatal plate which did not interfere with the occlusion. Consequently, subjects may have been impaired tongue movement especially in bolus formation and transportation during mastication immediately post-insertion. After their chewing ability was recovered by 7 days, brain activity in which is known to be associated with chewing may have fully recovered. This recovery suggests that adapting to palatal coverage may be associated with cortical activity changes in certain specific regions required to learn tongue motor control.

Some studies have indicated that the impairment of tongue movement may induce cortical adaptive changes. Cortical activity during a tongue motor task was found to be altered after a tongue motor disturbance, such as in the form of a partial glossectomy of the tongue^10,11^. Following surgery, increased activation was observed in the superior parietal lobule, supplementary motor area, and anterior cingulate cortex, which can be involved in the planning, execution, or control of the tongue motor task^11^. In addition, complex tongue motor performance may induce corticomotor plasticity which could be related to tongue motor control^12^. Thus, we hypothesized that palatal coverage may lead to a change of higher-order brain activity which can be associated with tongue motor regulation or motor planning, in addition to tongue motor execution. After the adaptation process, we presumed the improvement of tongue motor smoothness may be associated with decrease of brain activation in these areas. Here, we sought to investigate the neurophysiological mechanisms underlying the tongue movement adaptation process in response to an oral environmental change. In this study, we included the same subjects, and they wore the same palatal plate as our previous study^9^. To observe the association between behavioral tongue movement and brain activity, subjects performed the new tongue motor task to simplify the tongue movement of bolus formation or transportation.

## Methods

### Subjects

Twenty-eight healthy right-handed subjects with completely natural dentition participated in the study. We referred to the sample size in functional magnetic resonance imaging (fMRI) studies on human chewing (n = 17^13^, n = 29^14^). Students and clinical staff of Tokyo Medical and Dental University were recruited, who did not have any previous experience wearing removable dentures. Subjects who had experience wearing removable dentures, were undergoing orthodontic therapy, had symptoms of a temporomandibular disorder, psychiatric or neurologic disease, had a glossectomy of the tongue, or suffered from any tongue disease were excluded. Written informed consent was obtained from each subject after having explained the study aims and methodology. This study was approved by the Ethics Committee of Tokyo Medical and Dental University (Approval No. 1175, 1219) and conducted in accordance with the Declaration of Helsinki.

Subjects were asked to wear the palatal plate for 1 week non-stop except while sleeping. The resin plate which thickness was 3.0 mm was covered on the palate. The wrought wire clasps made of cobalt-chrome alloy were placed bilaterally on the patients’ second molars to retain the palatal plate. The design of the palatal plate has been described previously^9^. No susceptibility artifacts of the brain images were found with these clasps in the preliminary study. We assessed tongue motor dexterity, difficulty with tongue movement, and brain activity at pre-insertion (Day 0), immediately after insertion (Day 1), and at 3 (Day 3) and 7 days (Day 7) post-insertion.

### Tongue motor dexterity and difficulty with tongue movement

We designed a novel tongue motor performance task to measure tongue motor dexterity, which is simplified form of the bolus formation or transportation task and may be prevented after wearing the plate. We used a rubber tube which serves as an intermaxillary elastic in orthodontics (TOMY Inc, Tokyo, Japan). Subjects were asked to alternately move the rubber tube (diameter 10 mm, length 20 mm, weight 0.6 g) to either side of their mouth with their tongue, and bite into the tube with their molars. In total, they repeated this action 30 times. To reduce the learning effect of the tongue movement task itself during the experiment, we examined the number of adequate training sessions in which subjects were able to move their tongue rhythmically in the preliminary study. Subsequently, subjects were instructed to practice performing this whole exercise three times for one week until Day 0 in order to move their tongue rhythmically as much as possible. After they performed this exercise well and could move the tube rhythmically, an examiner recorded the time (s) required to perform 30 strokes of this tongue movement and calculated the tongue motor speed (strokes/s). The measurements were repeated six times separated by a one-minute interval. The average tongue motor speed obtained from the six trials was taken to represent tongue motor dexterity.

We also asked subjects to rate the difficulty of performing the tongue movement on a 100-mm visual analogue scale (VAS) on each experimental day. The anchor at the left end of the scale reflected a rating of “easy to move”, while that at the right end indicated “extremely difficult to move”. The distance (mm) from the left anchor to a vertical line drawn by the subject was taken to represent the score.

### Brain activity during tongue movement

We measured brain activity using fMRI (block design paradigm) during tongue movement. The task began with 18 s of rest followed by six sets of alternating 18 s tasks and 18 s rests. Subjects were asked to lie comfortably on the scanner table with their head immobilized with foam pads and straps around the forehead. Visual stimuli were projected onto a small screen attached to an MRI head coil. The visual stimulation method has been described previously^9^. During the rest period, a white fixation cross was displayed on the center of the black screen, and subjects were asked to relax their jaw and focus on the fixation point without moving their tongue. After the rest period, the cross disappeared and “tongue movement” in yellow letters was displayed on the screen. Subjects moved the tube with their tongue in the same way as in the behavioral experiment during the task period. Measurements were performed on Days 0, 1, 3, and 7.

fMRI data were acquired using a MAGNETOM Spectra 3 T scanner (Siemens, Erlangen, Germany) with a T2*-weighted gradient-echo echo-planar imaging (EPI) sequence (field of view 192 mm; matrix 64 × 64; 34 axial slices (thickness 3 mm, gap 0.75 mm) ; repetition time 2000 ms; echo time 30 ms; flip angle 77°; voxel size 3 mm × 3 mm × 3 mm). The initial nine volumes of each session were discarded to allow for magnetization stabilization. High-resolution T1-weighted images were acquired as anatomical references (field of view 250 mm; 192 sagittal slices (thickness 1 mm); repetition time 1900 ms; echo time 2.42 ms; voxel size 1 mm × 1 mm × 1 mm).

### Statistical analysis

#### Behavioral analysis

We performed statistical comparisons of behavioral data across time points using a linear mixed model analysis with Bonferroni corrections for multiple post-hoc comparisons. P-values < 0.05 were considered statistically significant. SPSS Statistics Version 23 (IBM, Tokyo, Japan) was used for all statistical analysis. The statistical analysis of behavioral data has also been described previously^9^.

#### Neuroimaging analysis

Images preprocessing and analyses were performed using Statistical Parametric Mapping software SPM12 v6685 (Wellcome Trust Centre for Neuroimaging, London, UK; https://www.fil.ion.ucl.ac.uk/spm/software/spm12/) implemented in Matlab (Mathworks, Natick, MA, USA). All functional scans were realigned to the first volume to correct for head movement and the mean of the realigned images was generated. We chose the middle time-point slice acquired at 1000 ms in each scan as the reference slice for the slice acquisition timing correction. The T1-weighted image was coregistered to the mean EPI image. The EPI image was transformed to the standard Montreal Neurological Institute (MNI) space. Functional data were normalized and spatially smoothed with an 8 mm full-width Gaussian kernel at half-maximum. At the first-level analysis, we constructed a general linear model for each subject on each experimental day with predictors for each task block modelled by canonical hemodynamic response function. A contrast image representing brain activation during the tongue movement task relative to baseline was generated in each individual analysis. Next, a within-subjects one-way ANOVA was performed on each contrast for second-level random-effects analyses. Statistical activation maps were computed for the tongue movement task relative to baseline. The threshold was set to p < 0.001 with correction for family-wise error (FWE). This neuroimaging analysis has also been described previously^9^. To compare brain activation patterns between pre- and post-plate insertion time points, and across post-insertion experimental days, a subtraction analysis was performed between statistical activation maps on Days 0 and 1, and between Days 1, 3, and 7, while using tongue motor speed as a covariate. The voxel-level threshold was set to p < 0.001 (uncorrected), and the cluster-level was set to p < 0.05 (uncorrected). Brain regions were anatomically defined and labelled according to the peak coordinate of a Talairach atlas^15^ after a non-linear transform of the MNI brain template to Talairach (http://imaging.mrc-cbu.cam.ac.uk/imaging/MniTalairach). Beta values from the regions of interest (ROIs) were extracted using Mars Bar software (http://marsbar.sourceforge.net/). The method of ROI analysis has also been described in our previous study^9^. As well as the behavioral analysis, we compared the beta-values from ROIs across time points using a linear mixed model analysis with Bonferroni corrections for multiple post-hoc comparisons. P-values < 0.05 were considered statistically significant.

## Results

Twenty-eight subjects (15 male, 13 female; mean age ± standard deviation: 28.6 ± 2.5-years-old) were enrolled in the study and completed all measurements. For fMRI measurements, one subject moved their head by more than > 1 voxel and the remaining 27 subjects were included for further analysis.

### Tongue motor dexterity and difficulty with tongue movement

**Figure 1:**
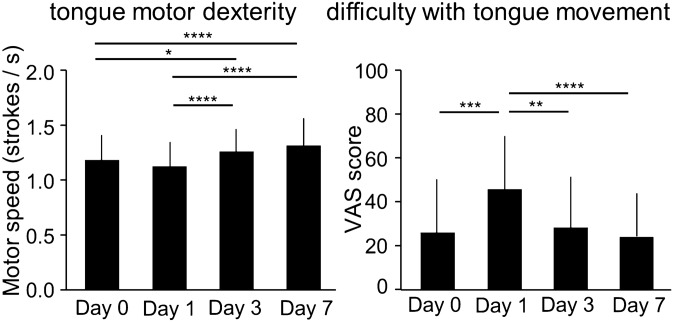
Change in tongue motor dexterity and difficulty of tongue movement (n = 27). Error bars represent standard deviation. Day 0: pre-insertion; Day 1: immediately after insertion; Day 3: 3 days after insertion; Day 7: 7 days after insertion. VAS: visual analogue scale. *P < 0.05, **P < 0.01, ***P < 0.005, **** P < 0.001.

The linear mixed model analysis found a significant time effect on tongue motor dexterity (p < 0.001). Post-hoc multiple comparisons found that the tongue motor dexterity on Day 3 was significantly higher than on Days 0 and 1 (p < 0.05), and that the dexterity on Day 7 was also significantly higher than on Days 0 and 1 (p < 0.05). Non-significant differences were observed between Days 0 and 1, and between Days 3 and 7. Difficulty with tongue movement also had a significant time effect (p < 0.001). Difficulty with tongue movement was significantly higher on Day 1 compared to Days 0, 3, and 7 (all ps < 0.01), and non-significant differences were observed between Day 0, Day 3, and Day 7 (Fig. 1).

### Brain activity

**Figure 2:**
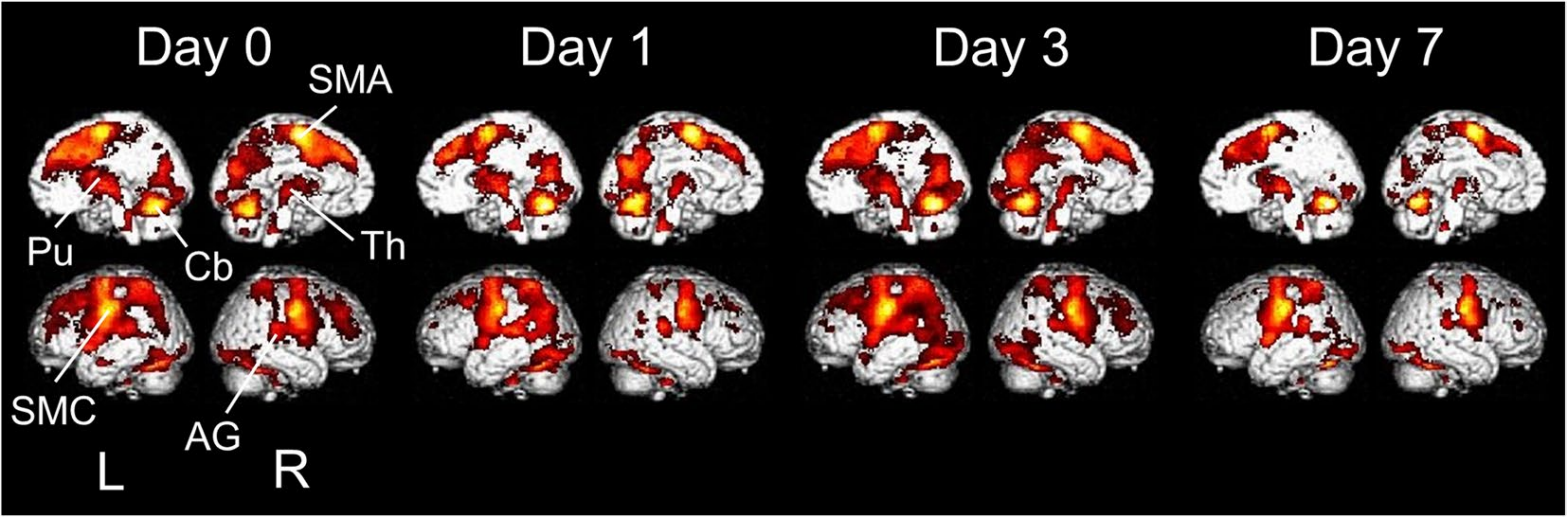
Activated areas during the tongue movement task projected onto the medial (top) and lateral (bottom) surfaces of the brain (n = 27) implemented in SPM12. L: left hemisphere; R: right hemisphere. SMC: sensorimotor cortex; Cb: cerebellum; Pu: putamen; Th: thalamus; SMA: supplementary motor area; AG: angular gyrus.

Figure 2 shows the significant activity above baseline during tongue movement on each experimental day (p < 0.001, FWE corrected). The sensorimotor cortex, cerebellum, putamen, thalamus, supplementary motor area, and angular gyrus were bilaterally activated at all time points.

**Figure 3:**
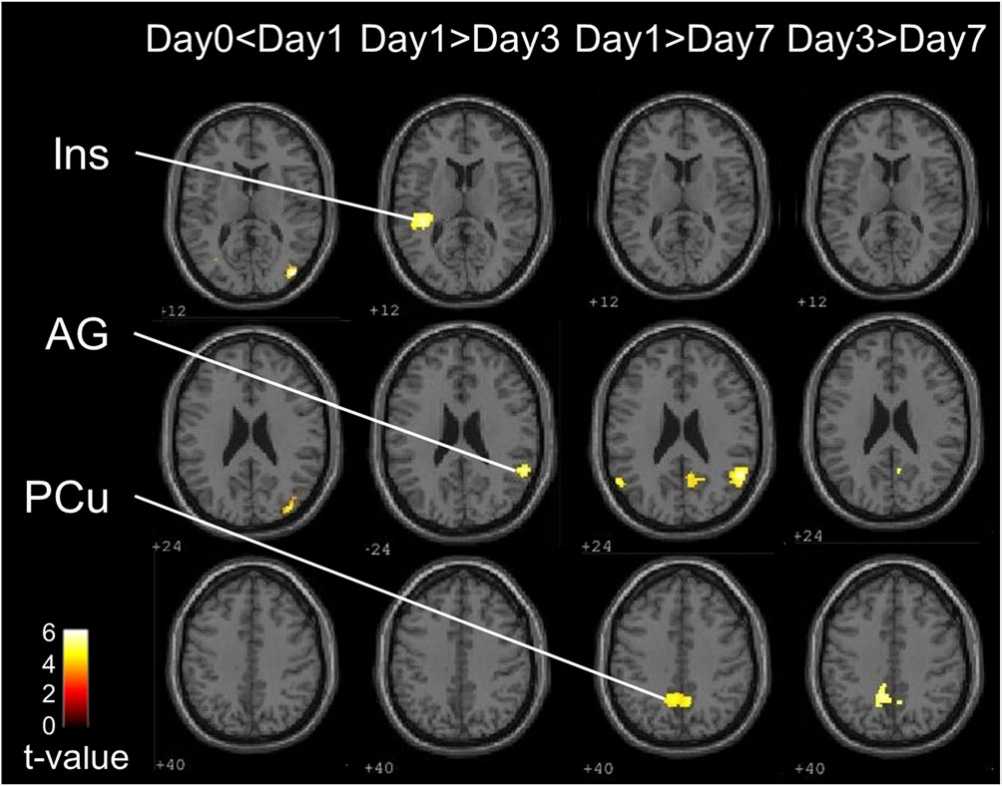
Brain regions significantly activated during tongue movement on Day 1 compared to Day 0, Day 3, and Day 7, and on Day 3 compared to Day 7 (n = 27) implemented in SPM12. Ins: insula; AG: angular gyrus; PCu: precuneus.

We compared the activation on Day 1, in which the tongue motor speed was the lowest, and when difficulty with tongue movement was the highest (higher than on Day 0). Brain activation in the bilateral middle temporal gyrus was significantly higher on Day 1 than on Day 0 (Table 1). In addition, we compared the brain activation patterns across post-insertion experimental days. On Day 1, compared to Day 3, the right angular gyrus and left insula were significantly activated. On Day 1, compared to Day 7, the bilateral angular gyrus and right precuneus were significantly activated. Finally, on Day 3, compared to Day 7, the bilateral precuneus was significantly activated (Table 1, Fig. 3).

**Table 1:** Significant clusters and their peak activations in the comparison between pre-insertion (Day 0) and immediately after insertion (Day 1), and between Days 1, 3, and 7 after insertion are listed.

Contrast	Region	Side	Cluster size	Peak statistics		Peak coordinates					
						MNI (mm)			Talairach (mm)		
				t	z	x	y	z	x	y	z
Day 0 < Day 1	Middle Temporal Gyrus	R	246	5.83	4.59	42	− 82	12	42	− 79	15
	Middle Temporal Gyrus	L	171	4.76	3.98	− 42	− 62	4	− 42	− 60	7
Day 1 > Day 3	Angular Gyrus	R	148	4.75	4.30	58	− 48	26	57	− 45	26
	Transverse Temporal Gyrus / Insula	L	306	4.55	4.15	− 36	− 34	12	− 36	− 32	13
Day 1 > Day 7	Angular Gyrus	R	265	4.85	4.38	54	− 54	24	53	− 51	25
	Precuneus	R	607	4.46	4.08	10	− 54	36	10	− 51	36
	Angular Gyrus	L	122	4.04	3.74	− 48	− 68	32	− 48	− 64	33
Day 3 > Day 7	Precuneus	R	165	4.09	3.79	10	− 50	34	10	− 47	34
	Precuneus	L	183	3.87	3.61	− 8	− 52	40	− 8	− 49	39

Brain regions were defined according to the peak coordinate of the Talairach atlas.

*MNI* montreal neurological institute.

**Figure 4:**
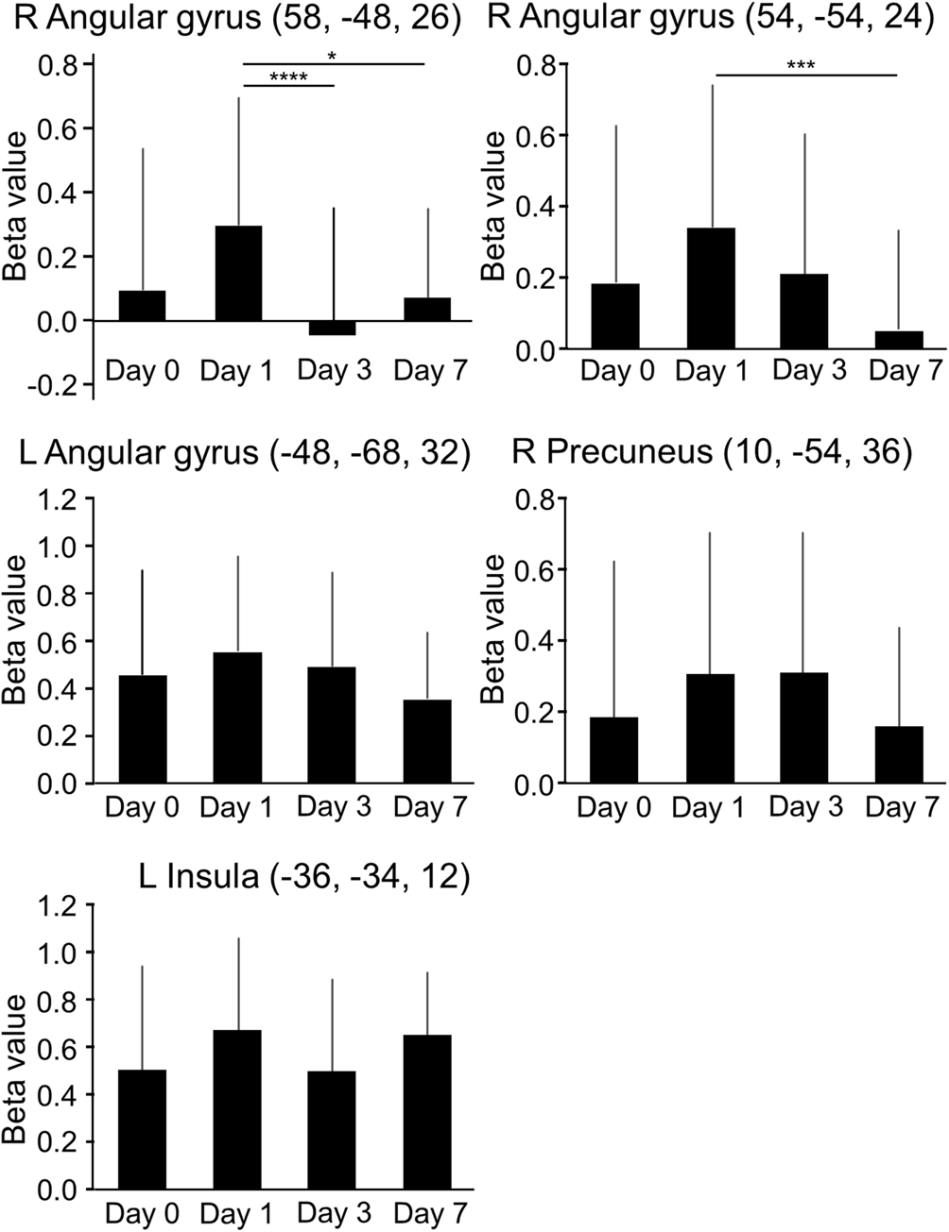
The beta values of the peak coordinates of the ROIs which were more significantly activated on Day 1 than on Day 3 or Day 7 (n = 27). Error bars represent standard deviation. ROI: region of interest. *P < 0.05, ***P < 0.005, ****P < 0.001.

We selected five regions which were more significantly activated on Day 1 than on Day 3 or 7 for the functional ROI analysis, and evaluated the temporal activity changes over the course of 7 days. All ROIs were defined in MNI space as peak coordinates of activity, as shown in Table 1: right angular gyrus and left insula (Day 1 > Day 3); right angular gyrus, right precuneus and left angular gyrus (Day 1 > Day 7). The beta values from these ROIs were extracted at each time point. The linear mixed model analysis using the same criteria as for the behavioral data found a significant time effect on the beta values in the ROIs of the right angular gyrus (p < 0.005) (Fig. 4). Post-hoc multiple comparisons revealed that the beta values of the right angular gyrus on Days 3 and 7 were significantly lower than on Day 1 (p < 0.05), whereas the left angular gyrus values were not significantly different across days. In the right precuneus and the left insula, non-significant differences in the beta values were observed between each day.

## Discussion

To the best of our knowledge, this is the first report on adaptive changes to palatal coverage in light of the relationship between tongue motor control and changes in brain activity. Immediately after palatal coverage, subjects found it difficult to move their tongue. Over the 7 days after plate insertion, tongue motor dexterity increased, and their difficulty with tongue movement decreased. To probe the mechanisms underlying adaptation to palatal coverage by assessing actual tongue movement, we designed a novel tongue motor task in the form of voluntarily controlling the movement of a bolus. Coordinated movement of the tongue, lips, jaws, and buccal mucosa may be required while performing this complex task. The brain areas activated above baseline while performing this tongue movement (Fig. 2) corresponded to the tongue motor areas indicated in other studies^16–19^. Especially in rhythmic isometric tongue movements, activation was observed in the putamen, thalamus, and cerebellum, which could be associated with voluntary control of movement, as well as in the primary sensorimotor cortex^17,19^.

In the subtraction analysis of brain activity across each day, brain activation in the bilateral angular gyrus and right precuneus immediately post-insertion was found to be significantly higher than at 7 days post-insertion. These results suggested that impaired tongue movement may activate the angular gyrus, which is associated with monitoring spatial information of the tongue, and activate the precuneus, which is associated with constructing the motor imagery of the tongue in response to a new oral environment. After the tongue fully recovered a certain degree of motor smoothness 7 days post-insertion, the angular gyrus and precuneus activation levels decreased. A previous fMRI study reported that the bilateral superior parietal lobule and the inferior parietal lobule were specifically involved in spatial processing in the context of human tongue movement^20^. The angular gyrus is located in the posterior part of the inferior parietal lobule and plays a role in spatial cognition^21^. In terms of the perceptual sequence learning mechanism, the right angular gyrus has been found to play a key role in the orienting of spatial attention^22^. The precuneus is located in the postero-medial portion of the parietal lobe and is involved in motor imagery^23^. In a PET study which revealed brain activity patterns in the context of motor imagery associated with locomotion, the bilateral precuneus was found to be significantly activated during a complex walking imagery task^24^. This study’s results indicated that the insertion of the plate changed the oral environment, which resulted in the disturbance of smooth tongue movement. Immediately post-insertion, the activation of angular gyrus may have been associated with monitoring spatial information related to the tube which had been placed in the oral region. In addition, the activation of the precuneus may have been associated with constructing the motor imagery of the tongue in response to this new oral environment. During the 7 days post-insertion, subjects could have moved the tube smoothly with their tongue without monitoring the oral information or constructing the motor imagery, leading to adaptation to palatal coverage. After having recovered tongue motor dexterity 7 days post-insertion, the activation levels of the angular gyrus and precuneus decreased. Therefore, for patients who cannot adapt to dentures, tongue motor training that helps to construct motor imagery of the tongue may be effective.

Subjects experienced difficulty moving their tongue immediately after palatal coverage, while tongue motor dexterity was nearly maintained at pre-insertion levels. We asked subjects to rhythmically move their tongue as much as possible, such that subjects would have had to make more effort on Day 1 than Day 0 to maintain this rhythm while wearing the plate. Meanwhile, tongue motor speed was higher at 7 days post-insertion than at pre-insertion. Subjects may have thus learned how to perform this rhythmical tongue movement in the new oral environment, as their tongues gradually felt increasingly easier to move leading up to Day 7. In addition, in the adaptation period, subjects may have easily moved the tube using their tongue, like while wearing the palatal augmentation prosthesis which enhanced lingual-palatal contact^25^.

It is difficult to evaluate tongue motor dexterity without directly assessing it with video fluoroscopy^26^, ultrasonography^27^, or MRI^3^. However, these measurements are limited by physical requirements or long examination times. Therefore, evaluating tongue movement performance in this study may be used for oral exercise training or dysphagia rehabilitation in the elderly, similar to the task in a previous study consisting of repeatedly rolling a ball to the right and left side of the mouth^16^.

There are some limitations to this study. First, there was no control group of subjects who did not wear the palatal plate for 7 days. Thus, the changes in brain activation patterns may have included both the adaptive effect to the new oral environment and the learning effect of the tongue movement task itself. In addition, we designed the palatal plate such as not to change the occlusion of the subjects. As such, it remains unknown whether adaptation to removable dentures is affected by a change in occlusion. Wearing the palatal plate may be a smaller intervention than wearing the removable dentures to investigate adaptive changes to a new oral environment. Moving the tube in the mouth may have caused an increase in frequent swallowing. This may have affected brain image quality. Finally, most of the individuals wearing dentures were elderly patients. In this study, we recruited fully dentate young participants to standardize the denture experience, but the duration or brain activation patterns in response to this adaptive change may be different between young and elderly participants.
